# Biomarkers of vascular injury in relation to myocardial infarction risk: A population-based study

**DOI:** 10.1038/s41598-018-38259-y

**Published:** 2019-02-28

**Authors:** Laura Pletsch-Borba, Mirja Grafetstätter, Anika Hüsing, Sandra González Maldonado, Manja Kloss, Marie-Luise Groß, Theron Johnson, Disorn Sookthai, Peter Bugert, Rudolf Kaaks, Tilman Kühn

**Affiliations:** 1 Division of Cancer Epidemiology, German Cancer Research Center (DKFZ), Heidelberg, Germany; 2 Medical Faculty, University of Heidelberg, Heidelberg, Germany; 3 Department of Neurology, University of Heidelberg, Heidelberg, Germany; 4 Institute of Transfusion Medicine and Immunology, Heidelberg University, Medical Faculty Mannheim, and German Red Cross Blood Service Baden-Württemberg-Hessen, Mannheim, Germany

**Keywords:** Diagnostic markers, Cardiology, Predictive markers, Epidemiology, Risk factors

## Abstract

Little is known about circulating biomarkers of vascular injury in relation to cardiovascular disease risk. Thus, we evaluated associations between six novel markers (E-Selectin, P-Selectin, thrombomodulin, thrombopoietin, intercellular adhesion molecule 3 and GPIIb/IIIa) and established cardiovascular risk factors as well as the risk of myocardial infarction (MI) in a population-based study. Biomarkers were measured in pre-diagnostic plasma samples of a case-cohort subset of EPIC-Heidelberg (incident MI cases: n = 369, random sub-cohort: n = 2,418). Generalized Linear models were used to analyse cross-sectional associations between biomarkers and cardiovascular risk factors. Multivariable Cox Regression analyses were carried out to obtain Hazard Ratios (HRs) of MI across quartiles of biomarkers levels. Cross-sectional analyses showed that sex, smoking, alcohol consumption, diabetes and exogenous hormone use were associated with biomarker levels. However, while fibrinogen was associated with MI risk (HR per standard deviation: 2.97 [95% confidence interval: 1.61, 5.46]), none of the six novel biomarkers was associated with MI risk after multivariable adjustment. In a population-based cohort, biomarkers of vascular injury were associated with established cardiovascular risk factors, but not MI risk. The tested biomarkers may reflect pathophysiological alterations in cardiovascular disease development rather than constituting independent MI risk factors.

## Introduction

Myocardial infraction (MI), the most common cardiovascular cause of death, is driven by atherosclerosis^1^. Vascular injury, one of the hallmarks of atherosclerosis, is mediated by complex interactions between the endothelium, activated platelets and immune cells that involve a plethora of signalling molecules such as inflammatory cytokines, growth factors, adhesion molecules, and coagulation factors^2–4^. While biomarkers of inflammation and coagulation have been assessed in large-scale epidemiological studies in relation to incident MI^5–7^, surprisingly little is known about platelet-derived signalling molecules and adhesion factors as potential biomarkers of MI risk^4^.

For the present prospective study on MI risk, we selected the following six circulating factors implicated in vascular injury and primary haemostasis as candidate biomarkers of MI risk: E-Selectin, P-Selectin, thrombomodulin (TM), thrombopoietin (TPO), intercellular adhesion molecule 3 (ICAM3), and glycoprotein IIb/IIIa (GPIIb/IIIa). Higher circulating levels of E-Selectin (mediating immune cell adhesion to the endothelium) and P-Selectin (mediating platelet adhesion) have been related to atherosclerosis and thrombosis^8–10^. GPIIb/IIIa is a receptor expressed on the surface of activated platelets, essential for platelet aggregation via binding to fibrinogen^11^. The molecule is a target in the treatment of acute coronary syndromes by GPIIb/IIIa inhibitors, but its platelet-derived circulating form has not been evaluated in large-scale epidemiological studies^12^. TM is a membrane glycoprotein expressed by endothelial cells that converts thrombin from a procoagulant to an anticoagulant enzyme, thus modulating platelet activation and haemostasis^13^. TPO is a humoral substance produced by the liver responsible for stimulating platelet production^14^. As a circulating biomarker, it has been shown to be inversely associated with biomarkers of platelet turnover^15^. Finally, ICAM3 is an integrin that may mediate interactions between leucocytes and endothelial cells in atherosclerosis^16,17^.

Given the lack of epidemiological studies on the above-mentioned plasma markers, we used the population-based EPIC-Heidelberg cohort to evaluate associations (a) between the markers and established cardiovascular risk factors in cross-sectional analyses, and (b) between the markers and MI risk in prospective analyses. In addition, we carried out mediation analyses to investigate whether relationships between established cardiovascular risk factors and MI risk could be mediated by the proposed biomarkers.

## Results

### Characteristics of the study population

Median baseline age in the sub-cohort was 51.0 (IQR 14.1) years, and 53% were women, while among MI cases mean age was 56.7 (IQR 10.1) years, and 22% were women. Baseline values for cardiovascular risk factors and biomarkers among cases and in the sub-cohort are reported in Table 1. Median follow-up of the sub-cohort and the MI cases was respectively 9.8 (IQR 1.9) and 6.1 (IQR 4.6) years. Cases showed a worse cardiovascular risk profile than the participants in the sub-cohort. Aspirin use was reported by 7.0% of the cases and 3.4% of the individuals in the sub-cohort. The prevalence of antithrombotic medication use beyond aspirin at baseline was 0.5% among cases and 0.4% in the sub-cohort.

**Table 1:** Baseline characteristics of the EPIC-Heidelberg Case-Cohort Sample (1994–1998).

	MI Cases	Sub-cohort		
	Total (n = 369)	Women (n = 1293, 53%)	Men (n = 1125, 47%)	Total (n = 2418)
Age at recruitment (years)^a^	56.7 (10.1)	48.59 (15.4)	53.05 (11.8)	51.03 (14.1)
Age at diagnosis of MI (years)^a^	60.6 (10.6)	58.7 (15.0)	62.6 (12.2)	60.6 (13.6)
Hypertension (yes)^b^	165 (44.7%)	303 (23.4%)	375 (33.3%)	678 (28.0%)
Antihypertensive drug use (yes)^b^	137 (37.1%)	253 (19.6%)	279 (24.8%)	352 (22.0%)
Diabetes mellitus (yes)^b^	65 (17.6%)	28 (2.2%)	64 (5.7%)	92 (3.8%)
Body mass index (kg/m²)^a^	27.6 (5.5)	24.3 (5.9)	26.3 (4.6)	25.5 (5.5)
Height (cm)^a^	172.2 (12.8)	163.6 (8.3)	175.9 (8.4)	169 (12.8)
Weight (kg)^a^	82 (19.9)	65.5 (16.1)	81.8 (8.4)	74 (19.9)
Waist circumference (cm)^a^	96.7 (19.5)	79 (16.0)	94.7 (13.5)	88 (19.5)
Alcohol intake at Baseline (g/day)^a^	10.6 (21.6)	4.3 (8.1)	19.7 (29.4)	10.2 (21.6)
Education level^b^				
Primary School ^b^	153 (41.5%)	350 (27.1%)	334 (29.7%)	684 (28.3%)
Secondary School^b^	130 (35.2%)	625 (48.3%)	363 (32.3%)	988 (40.9%)
University Degree^b^	86 (23.3%)	318 (24.6%)	428 (38.0%)	746 (30.9%)
Smoking Status^b^				
Never	122 (33.1%)	657 (58.4%)	381 (33.9%)	1038 (42.9%)
Former, stopped <10 yrs ago^b^	77 (20.9%)	234 (20.8%)	314 (27.9%)	548 (22.7%)
Former, stopped ≥10 yrs ago^b^	36 (9.8%)	131 (11.6%)	131 (11.6%)	266 (11.0%)
Current less than 15 cigarettes/day^b^	60 (16.3%)	175 (15.6%)	175 (15.6%)	312 (12.9%)
Current ≥than 15 cigarettes/day^b^	74 (20.1%)	96 (8.5%)	96 (8.5%)	254 (10.5%)
Aspirin use (yes)^b^	26 (7.1%)	32 (2.5%)	49 (4.4%)	81 (3.4%)
Antithrombotic drug use (yes)^bc^	2 (0.5%)	7 (0.5%)	2 (0.2%)	9 (0.4%)
Physical Activity^b^ (Cambridge index)				
Inactive/moderately inactive ^b^	185 (50.1%)	623 (48.2%)	503 (44.7%)	1126 (46.6%)
Moderately active/active ^b^	184 (49.9%)	670 (51.2%)	622 (55.3%)	1292 (53.4%)
CRP (mg/l)^a^	2.0 (2.9)	0.95 (2.0)	1.1 (2.1)	0.99 (2.1)
LDL (mmol/l)^a^	4.5 (1.4)	3.8 (1.4)	4.1 (1.3)	4.0 (1.4)
Tryglicerides (mmol/l)^a^	2.3 (1.9)	1.3 (1.0)	1.9 (1.4)	1.6 (1.2)
HDL(mmol/l)^a^	1.2 (0.4)	1.6 (0.6)	1.2 (0.5)	1.4 (0.6)
Total Cholesterol (mmol/l)^a^	6.2 (1.4)	5.8 (1.4)	5.8 (1.4)	5.8 (1.4)
Fibrinogen (µg/ml)^a^	4050.8 (933.6)	3783.4 (893.9)	3759.6 (849.0)	3775.3 (876.3)
E-Selectin (ng/ml)^a^	11.2 (6.6)	9.2 (5.7)	11.1 (6.6)	9.9 (6.4)
P-Selectin (ng/ml)^a^	30.6 (13.5)	25.7 (11.2)	30.2 (13.2)	27.5 (12.4)
Thrombomodulin (ng/ml)^a^	3.0 (0.9)	2.7 (0.9)	3.06 (1.0)	2.9 (1.0)
Thombopoietin (pg/ml)^a^	340.3 (125.2)	350.1 (125.1)	332.4 (117.3)	340.4 (122.4)
ICAM3 (ng/ml)^a^	0.47 (0.19)	0.43 (0.19)	0.46 (0.20)	0.44 (0.19)
Glycoprotein IIb/IIIa (ng/ml)^a^	384.0 (198.3)	384.0 (182.3)	382.1 (176.3)	382.8 (179.5)

Data presented as ^a^median (interquartile range) and ^b^number (proportion). ^c^Antithrombotic drugs excluding aspirin. CRP, C-reactive protein; LDL, low-density lipoprotein; HDL, high-density lipoprotein; ICAM3, intercellular adhesion molecule 3.

### Cross-sectional associations between biomarkers and cardiovascular risk factors

**Figure 1:**
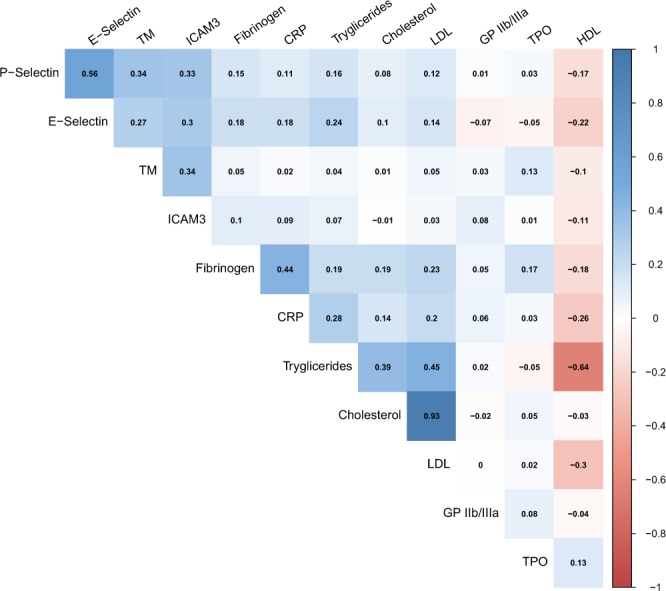
Cross-sectional correlation matrix between plasma biomarkers and lipid levels. Spearman’s correlations between plasma biomarkers. TM indicates thrombomodulin, ICAM3 intercellular adhesion molecule III, CRP C-reactive protein, LDL low-density lipoprotein, GPIIb/IIIa glycoprotein IIb/IIIa, TPO thrombopoietin, and HDL high-density lipoprotein.

Figure 1 depicts Spearman’s coefficients (ρ) for correlations among biomarkers. P-Selectin showed correlations with E-Selectin, ICAM3 and TM at Spearman’s coefficients of 0.56, 0.33, and 0.34. E-Selectin showed weak correlations with ICAM3 and TM (ρ = 0.30 and ρ = 0.27). Supplementary Table 1 show multivariable adjusted cross-sectional associations between biomarkers of vascular injury and established cardiovascular risk factors. Women presented lower levels of E-Selectin, P-Selectin, and TM than men. Smokers had increased levels of P-Selectin. Heavier alcohol drinkers had increased levels of E-Selectin, but lower levels of TM and TPO. Individuals with diabetes at baseline had higher levels of E-Selectin, P-Selectin and ICAM3. Among women, users of oral contraceptives and hormone replacement therapy at baseline revealed lower levels of E-Selectin, P-Selectin, ICAM3, TM, and TPO. A longer duration of oral contraceptives use was inversely associated with TM, while a longer duration of hormone replacement therapy was inversely associated with P-Selectin and E-Selectin levels. As fibrinogen, the established marker of coagulation, was associated with a majority of cardiovascular risk factors, we further adjusted the analyses on E-Selectin, P-Selectin, TM, TPO, ICAM3, and GPIIb/IIIa for fibrinogen. However, adjustment for fibrinogen only led to marginal changes of the described associations (see Supplementary Table 2).

### Associations between Biomarkers and Risk of Myocardial Infarction

**Figure 2:**
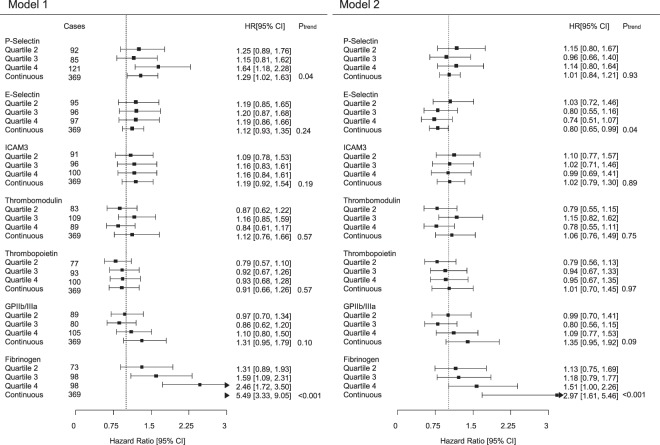
Forest plot of associations between plasma biomarkers and myocardial infarction risk. Associations between plasma biomarkers and myocardial infarction risk. Left plots illustrate results from model 1 (age- and sex-adjusted). Right plots illustrate results from model 2, additionally adjusted for height (m), waist circumference (cm), alcohol consumption (g/day in the past year), smoking status (never, former quitted > 10 y ago, former quitted ≤10 y ago, current <15 cigarettes/day, current ≥15 cigarettes/day), hypertension, antihypertensive drug use, diabetes mellitus, C-Reactive Protein (CRP), total cholesterol, HDL cholesterol, education level, and physical activity level (Cambridge index). CI: confidence interval; Ptrend denotes the p-value from Cox regression analyses with biomarker levels on the continuous log2 standardized (mean = 0, SD = 1) scale.

From the baseline examination of EPIC-Heidelberg (1994–1998) until December 31^st^ 2006, 369 participants experienced a primary MI, out of which 27 (7%) were fatal (death within the 28 subsequent days of the MI). Associations between the biomarkers and MI risk are shown in Fig. 2. P-Selectin was positively associated with MI risk in model 1 (HR_highest vs lowest quartile_[95% CI]: 1.64 [1.18, 2.28]). However, upon multivariable adjustment, this association was no longer statistically significant (HR 1.14 [0.80, 1.64]). E-Selectin was associated with decreased MI risk after multivariable adjustment (HR per SD log2 E-Selectin [95% CI]: 0.80 [0.65, 0.99]), but not in model 1, and there was a significant association between fibrinogen, the routine marker of coagulation, and MI risk (HR: 2.97 [1.61, 5.46]). TM, TPO, GPIIb/IIIa, and ICAM3 were not significantly associated with MI risk. Interactions between biomarker levels with sex and age were not statistically significant; therefore stratified analyses were not carried out. Further adjustment of the multivariable statistical model for the use of aspirin and other antithrombotic drugs only marginally changed the HRs for MI (data not shown). Sensitivity analyses restricting the follow-up time to the first 5 years after baseline showed highly similar results compared with the main analyses covering the entire follow-up duration (Supplementary Table 3).

Mediation analyses based on the method proposed by VanderWeele^18^ were conducted for biomarkers with indications of associations with MI risk in model 1 at least, i.e. P-Selectin and fibrinogen (see Table 2). Multivariable analyses did not indicate that associations between lifestyle factors and MI risk were mediated via P-Selectin. By contrast, associations between smoking, alcohol consumption, and BMI and MI risk were partially mediated by plasma levels of fibrinogen (fully adjusted mediated proportions, respectively: 5.7%, p = 0.01; 15.7%, p < 0.001; 24.8%, p < 0.001).

**Table 2:** Direct and indirect (i.e. partially mediated via fibrinogen and P-Selectin) associations between cardiovascular risk factors and incident myocardial infarction.

	Direct effect		Indirect effect		Proportion mediated
	HR (95%CI)	P-value	HR (95%CI)	P-value	
**P-Selectin**					
*Smoking Status* ^*a*^					
Model 1	1.10(1.06,1,14)	<0.001	1.00(0.99,1.01)	0.1	4.3%
Model 2	1.09(1.05,1.13)	<0.001	1.00(0.99,1.01)	0.9	0.4%
*Current Smoking*					
Model 1	1.18(1.04,1.33)	<0.01	1.01(1.00,1.02)	0.049	7.7%
Model 2	1.00(0.99,1.00)	0.1	1.00(0.99,1.01)	0.7	1.5%
*Ever Smoking*					
Model 1	1.15(1.04,1.26)	<0.01	1.01(1.00,1.02)	0.04	7.9%
Model 2	1.12(1.02,1.24)	0.02	1.00(0.99,1.01)	0.7	1.7%
*Alcohol Intake* ^*b*^					
Model 1	0.96(0.93,0.98)	<0.01	1.01(1.01,1.02)	0.6	0.8%
Model 2	0.96(0.93,0.99)	0.02	1.00(0.99,1.00)	0.8	0.0%
*Prevalent Hypertension*					
Model 1	1.20(1.09,1.32)	<0.001	1.00(0.99,1.01)	0.8	0.4%
Model 2	1.12(0.96,1.29)	0.1	1.00(0.99,1.00)	0.9	0.2%
*Antihypertensive drug use*					
Model 1	1.18(1.07,1.29)	<0.01	0.99(0.99,1.01)	0.7	0.9%
Model 2	0.98(0.85,1.14)	0.8	0.99(0.99,1.00)	0.9	0.7%
*Prevalent diabetes mellitus*					
Model 1	1.35(1.18,1.54)	<0.001	1.00(0.99,1.01)	0.1	5.6%
Model 2	1.39(1.15,1.68)	<0.01	1.00(0.98,1.02)	0.8	0.3%
*BMI*					
Model 1	1.03(1.02,1.04)	<0.001	1.00(1.00,1.00)	0.06	2.7%
Model 2	1.03(0.95,1.10)	0.5	1.00(0.99,1.00)	0.9	0.1%
**Fibrinogen**					
*Smoking Status* ^*a*^					
Model 1	1.09(1.05,1.12)	<0.001	1.01(1.01,1.02)	<0.001	15.0%
Model 2	1.09(1.05,1.31)	<0.001	1.01(1.00,1.01)	0.01	5.7%
*Current Smoking*					
Model 1	1.16(1.02,1.31)	0.01	1.01(0.99,1.03)	0.02	9.4%
Model 2	1.18(1.05,1.33)	<0.01	1.01(0.99,1.02)	0.2	5.9%
*Ever Smoking*					
Model 1	1.13(1.03,1.25)	0.01	1.02(1.00,1.03)	0.01	13.4%
Model 2	1.12(1.02,1.24)	0.02	1.00(0.99,1.01)	0.5	3.4%
*Alcohol Intake* ^*b*^					
Model 1	0.96(0.94,0.98)	<0.01	0.99(0.99,0.99)	<0.01	20.1%
Model 2	0.97(0.94,0.99)	0.02	0.99(0.99,0.99)	<0.01	15.7%
*Prevalent Hypertension*					
Model 1	1.16(1.06,1.28)	<0.01	1.05(1.03,1.07)	<0.01	27.6%
Model 2	1.12(0.97,1.29)	0.1	1.01(0.99,1.02)	0.2	8.4%
*Antihypertensive drug use*					
Model 1	1.13(1.02,1.25)	0.02	1.05(1.03,1.07)	0.02	30.5%
Model 2	1.03(0.84,1.26)	0.8	1.01(0.97,1.06)	0.5	18.6%
*Prevalent diabetes mellitus*					
Model 1	1.47(1.29,1.68)	<0.001	1.09(1.05,1.12)	<0.001	25.9%
Model 2	1.37(1.17,1.61)	<0.001	1.03(0.97,1.10)	0.4	7.5%
*BMI*					
Model 1	1.02(1.01,1.03)	<0.001	1.01(1.01,1.01)	<0.001	29.3%
Model 2	1.01(1.00,1.02)	0.05	1.01(1.01,1.01)	<0.001	24.8%

^1^Categorized in never smoker, former quitted > 10 y ago, former quitted ≤10 y ago, current <15 cigarettes/day, current ≥15 cigarettes/day, and modelled as continuous trend variable. ^2^Categorized in no intake, ≤12 g/d, ≥12–23.9 g/d, ≥24–39.9 g/d, ≥40 g/d in the 1-year period previous to the interview, and modelled as continuous trend variable. Hazard Ratios (95% Confidence Intervals) for direct and indirect effects. Model 1 adjusted for age and sex. Model 2 additionally adjusted for height (m), waist circumference (cm), alcohol consumption (g/day), smoking status (never, former quitted > 10 y ago, former quitted ≤10 y ago, current <15 cigarettes/day, current ≥15 cigarettes/day), hypertension, antihypertensive drug use, diabetes mellitus, C-Reactive Protein (CRP), total cholesterol, HDL cholesterol, education level, physical activity level and P-Selectin/Fibrinogen when appropriate.

## Discussion

In the present study we found that, in a population-based cohort, biomarkers of vascular injury (P-Selectin, E-Selectin, GPIIb/IIIa, TPO, TM, and ICAM3) were independently associated with several established cardiovascular risk factors. However, in contrast to fibrinogen (the established risk marker of vascular injury and coagulation), none of these biomarkers was significantly associated with MI risk in longitudinal analyses. Mediation analyses did not suggest that associations between established CVD risk factors and MI risk could be mediated by the six biomarkers, whereas they did indicate that fibrinogen partially mediates the associations between smoking, alcohol consumption, and BMI with MI risk. Thus, E-Selectin, P-Selectin, TPO, TM, GPIIb/IIIa, and ICAM3 may reflect established CVD risk factors rather than constituting independent risk factors for MI.

As stated above, biomarkers of vascular injury showed several associations with cardiovascular risk factors in our cross-sectional analyses, independent of fibrinogen. In line with previous analyses from the KORA Study, our results suggested higher levels of E-Selectin among persons with prevalent diabetes^19,20^. While a significant inverse association between TM and incident diabetes was observed in the KORA Study, TM was neither associated with prevalent diabetes in our study nor in the KORA Study^20,21^. In our study, prevalent diabetes was significantly associated with increased P-Selectin levels, which is in line with a previous study^22^, and with ICAM3 levels, which has not been assessed before. The association between P-Selectin and smoking that we report has already been described, although not from multivariable models adjusted for other cardiovascular risk factors and fibrinogen^22^. Similarly, while a positive association between alcohol consumption and increased E-Selectin levels has been observed before^20^, we demonstrated that this association is independent of fibrinogen and other cardiovascular risk factors. This observation seems particularly interesting considering that alcohol was inversely associated with fibrinogen in our study - as in many others before^23^ - but also with GPIIb/IIIa and TPO, which may explain putative cardio-protective effects of alcohol intake^24^. Nevertheless, the positive association between alcohol intake and E-Selectin in our analyses suggests that alcohol intake may also induce pro-coagulant haemostatic factors. Such differential effects of alcohol may be one explanation for differential associations between alcohol intake and specific cardiovascular outcomes^25^. With regard to the use of hormones among women, the associations we found between oral contraceptive use and hormone replacement therapy with lower fibrinogen and E-Selectin levels, respectively, are again in line with previous findings^20,26^. Exogenous hormone use was further associated with lower levels of P-Selectin, TM, TPO, and ICAM3 in the EPIC-Heidelberg Study, which is consistent with potential anti-thrombotic and vasodilatory effects of exogenous hormones^27^, although a higher risk of MI among users of oral contraceptives has been reported^28^. Yet, we did not observe significant associations between exogenous hormone use or the biomarkers and MI risk in our study.

Given the range of significant associations between cardiovascular risk factors and the biomarkers of vascular injury outlined above, we carried out mediation analyses to investigate whether associations between established CVD risk factors and MI risk could be mediated by the biomarkers. While the mediation analyses using the method proposed by VanderWeele indicated no significant mediation effect of P-Selectin in the associations between cardiovascular risk factors and MI risk, our results suggest that fibrinogen may mediate the relationships between smoking, alcohol consumption, and BMI and MI risk. Overall, none of the six tested biomarkers in addition to fibrinogen in our study was significantly associated with MI risk over time upon multivariable adjustment. We did observe a borderline significant inverse association between E-Selectin levels on the log2 scale and MI risk that was only present upon multivariate adjustment. However, there were no significant differences between extreme quartiles, and we cannot rule out that the observed linear trend is a statistical artefact, since other previous studies have reported no associations between this biomarker and MI risk^29^. Thus, the markers may reflect haemostatic alterations due to known CVD risk factors, but do not seem to exert independent or indirect pathophysiological effects in MI development.

Our study had several limitations. We measured plasma biomarkers from blood samples obtained at a single time point, i.e. at the baseline of the EPIC-Heidelberg Study. However, all biomarkers had shown good biological reproducibility prior to the present analyses^30^, and regression dilution may not explain the lack of longitudinal associations with MI risk. Although our analyses have shown several statistically significant associations between biomarkers of vascular injury and established cardiovascular risk factors, the magnitude of most associations was rather low. Another limitation was that baseline data on the type of exogenous hormones used and measured blood pressure was not available for our analyses. Due to the lack of intact platelets, we did not have the opportunity to obtain routine parameters of platelet function and turnover, which would have been of interest in the present study. Lastly, the EPIC-Heidelberg cohort consists of rather health-conscious individuals compared to the German general population, and associations between biomarkers and CVD risk factors may be more pronounced in populations including participants with more unfavourable risk factor profiles, even though our sample did include meaningful proportions of heavy smokers, drinkers etc. to guarantee sufficient contrast for etiological analyses. The strengths of this study comprise the relatively large number of MI cases, the high quality and specificity (MI and not CHD) of case ascertainment, the prospective population-based set-up, the comprehensive set of biomarkers, and the wide range of available covariates.

## Conclusion

Taken together, our results suggest that biomarkers of vascular injury and platelet activation (E-Selectin, P-Selectin, TPO, TM, ICAM3, and GPIIb/IIIa) are associated with established CVD risk factors, particularly sex, alcohol consumption, diabetes, and exogenous hormone use. At the same time, they may not constitute independent risk factors for MI.

## Methods

### Study population

The EPIC-Heidelberg cohort included 25,540 participants aged from 35–65 years, who were recruited between 1994–1998 from the local general population^31,32^. At baseline, detailed questionnaire and interview assessments on diet, lifestyle factors, drug use (including oral contraceptive use and hormone replacement therapy after menopause), and health status were carried out, and anthropometric measurements were taken. Moreover, blood samples were collected, processed and stored for later measurement of biomarkers. Participants have been followed-up by active and passive procedures since baseline^31^. The Ethics Committee of the Heidelberg University Hospital approved the study and all participants gave written informed consent. All methods were performed in accordance with the relevant guidelines and regulations. The present analyses on plasma biomarkers and MI risk were conducted using a case-cohort data analysis design^33^. The study population consisted of a random sub-cohort (n = 2,418) and all incident cases of MI (n = 369) that occurred until December 31^st^ 2006 (Supplementary Fig. 1). From the n = 598 primarily identified incident cases of MI, 413 were validated (n = 185 falsified cases), n = 35 were excluded due to unavailability of blood samples, and n = 9 were excluded due to prevalent stroke at baseline.

### Ascertainment of Incident Myocardial Infarction

Incident cases of MI were identified based on self-reports from questionnaires (during three waves of follow-up, with response rates of > 95%), death certificates obtained via linkages with mortality registries, and record linkages with local university hospitals^34^. All cases were then validated by a trained study physician (MLG) using patient records. For 311 (84.3%) of the cases, a definitive validation by diagnostic records based on criteria of the World Health Organization, i.e. clinical presentation, electrocardiogram and cardiac biomarkers^35^ was possible, while 58 cases (15.7%) could only be validated by records of general practitioners, which did not include diagnostic details (see Supplementary Fig. 1). An exclusion of these 58 cases from statistical models only very marginally affected the results (data not shown), which was why we decided to include them in our risk analyses. The international classification of diseases (ICD-10) system was used to classify incident MI (ICD-10 I21).

### Laboratory Methods

Baseline blood samples were processed in citrate plasma, buffy coat, erythrocyte and serum samples, which were then aliquoted into plastic straws and stored in liquid nitrogen (−196 °C). A single plasma straw per participant was retrieved and stored in freezers at −80 °C for four weeks between samples retrieval and biomarker measurements. Aliquots were thawed prior to analyses of E-Selectin, P-Selectin, TM, TPO, ICAM3, GPIIb/IIIa, and fibrinogen. The “Quiplex SQ 120” instrument from Meso Scale Discoveries (MSD Maryland, USA) was used to measure E-Selectin, P-Selectin, TM, and ICAM3 (“human vascular injury kit I” multiplex assay kit from MSD) as well as TPO (“U-Plex TPO Assay” kit from MSD) by electrochemoluminescence immunoessays (ECLIA). GPIIb/IIIa and fibrinogen levels were measured by enzyme-linked immunosorbent assays (ELISA) using the essay kits “ab108851” from Abcam (Cambridge, UK) and “KA0475” from Abnova (Heidelberg, Germany). Kits came all from the same production lots. Samples from cases and non-cases were randomly assigned to the batches to minimize possible differential misclassification, and case status was blinded to the laboratory personnel. Each batch contained two quality control (QC) plasma samples in duplicate in order to monitor the validity of the measurements within and across the batches. Within-batch coefficients of variation (CVs) [between batch CVs] were 3.6% [10.6%] for E-Selectin, 3.3% [9.1%] for P-Selectin, 3.8% [10.1%] for TM, 4.6%[19.5%] for TPO, 7.5% [20.1%] for ICAM3, 5.5% [46.9%] for GPIIb/IIIa, and 5.7% [8.5%] for fibrinogen. Batch-standardisation of biomarker levels for multivariable regression analyses (see statistical analyses section) was performed by using the batch mean-centering method^36^. However, given the random allocation of samples from cases and non-cases on analytical batches, statistical analyses based on batch-standardized and non-batch-standardized biomarker levels showed highly similar results (data not shown).

Before the start of the present project, we tested the biological reproducibility of biomarker concentrations over one year in a sub-sample of 78 EPIC-Heidelberg participants, who were invited to the study center for a sub-study on body composition between 2010 and 2013. All biomarkers showed a good one-year reproducibility, with Spearman’s correlation coefficients of 0.80 (P-Selectin), 0.73 (TPO) 0.63 (TM), and 0.51 (GPIIb/IIIa)^30^. For the present study we further tested the one-year reproducibility of E-Selectin and ICAM3 in the same sub-sample, and obtained Spearman’s correlation coefficients of 0.88 for E-Selectin and 0.69 for ICAM3.

### Statistical Analyses

Continuous variables are described as median (interquartile range, IQR) and categorical variables as numbers (percentages). All plasma batch-standardized biomarkers were log2 transformed to better approximate a normal distribution, and standardized (mean of 0 and standard deviation [SD] of 1), in order to facilitate comparisons of hazard ratios per SD across biomarkers. We further categorized batch-standardized biomarker levels by quartiles (using cut-off points based on the sex-specific distribution in the sub-cohort) for cross-sectional and longitudinal regression analyses. Correlations between the biomarkers were evaluated by Spearman’s coefficients. Cross-sectional associations between biomarkers and categorical covariates were evaluated by Generalized Linear Models adjusted for age and sex (model 1) and additionally adjusting for established cardiovascular risk factors (model 2). The maximum percentage of missing values in the covariates was 7%. These were multiply imputed under the missing at random (MAR) assumption, using fully conditional (FCS) algorithm, with the number of imputations set to 5.

We used Cox proportional hazards regression models with the weighting method for case-cohort designs as suggested by Prentice to calculate hazard rate ratios (HR) and 95% confidence intervals (CI)^37^. Each plasma biomarker was analysed as an individual risk factor for incident MI. We left-truncated at the time of blood sampling, and all participants were censored at the time of MI diagnosis, death, loss of follow-up, or the end of the study follow-up (31^st^ December 2006), whichever occurred first. Covariates were chosen based on literature review. Model 1 was adjusted for age and sex. Model 2 was additionally adjusted for other cardiovascular risk factors i.e. height (cm), waist circumference (cm), alcohol consumption (g/day in the 1-year period before baseline), smoking status (never, past quitted ≥10 years ago, quitted < 10 years ago, current > 15 cigarettes/day, ≥15 cigarettes/day), hypertension (yes/no), antihypertensive drug use (yes/no), diabetes mellitus (yes/no), C-Reactive Protein (CRP), total cholesterol, HDL-cholesterol, education (primary, secondary, university), and physical activity (Cambridge Index; inactive/moderately inactive and moderately inactive/active)^38^. Extended correlation tests based on Schoenfield residuals indicated no violations of the proportional hazards assumption. Potential effect modifications of sex and age were tested including a multiplicative term into the Cox regression models. Furthermore, we used the method proposed by VanderWeele to perform statistical analyses on causal mediation^18^. A two-sided p-value of less than 0.05 denoted statistical significance, and statistical analyses were conducted in R (version 3.4.3) and SAS 9.4 (Cary, NC) for Windows.

## Supplementary information

Supplementary Tables

Electronic Supplementary Material

## Data Availability

Data are made available by the corresponding author upon reasonable request.
