# Author Correction: Biomarkers of vascular injury in relation to myocardial infarction risk: A population-based study

**DOI:** 10.1038/s41598-019-44430-w

**Published:** 2019-05-29

**Authors:** Laura Pletsch-Borba, Mirja Grafetstätter, Anika Hüsing, Sandra González Maldonado, Manja Kloss, Marie-Luise Groß, Theron Johnson, Disorn Sookthai, Peter Bugert, Rudolf Kaaks, Tilman Kühn

**Affiliations:** 1 Division of Cancer Epidemiology, German Cancer Research Center (DKFZ), Heidelberg, Germany; 2 Medical Faculty, University of Heidelberg, Heidelberg, Germany; 3 Department of Neurology, University of Heidelberg, Heidelberg, Germany; 4 Institute of Transfusion Medicine and Immunology, Heidelberg University, Medical Faculty Mannheim, and German Red Cross Blood Service Baden-Württemberg-Hessen, Mannheim, Germany

Correction to: *Scientific Reports* 10.1038/s41598-018-38259-y, published online 28 February 2019

A supplementary file containing Supplementary Figure 1 was omitted from the original version of this Article. This has been corrected in the HTML version of the Article; the PDF version was correct at time of publication.

