# Supplementary material for: Biomarkers of vascular injury in relation to myocardial infarction risk: A population-based study
Source: Sci Rep. 2019 Feb 28;9:3004. doi: 10.1038/s41598-018-38259-y (PMC6395643; doi:10.1038/s41598-018-38259-y)
Supplement: Supplementary file 2 — Electronic Supplementary Material [file 41598_2018_38259_MOESM2_ESM.docx]

**SUPPLEMENTARY MATERIAL**

**Biomarkers of vascular injury in relation to myocardial infarction risk:**

**A population-based study**

Laura Pletsch-Borba^1,2^, Mirja E. Graf^1^, Anika Hüsing^1^, Theron Johnson^1^, Sandra González Maldonado^1^, Manja Kloss^3^, Marie-Luise Groß^1^, Peter Bugert^4^, Rudolf Kaaks^1^, and Tilman Kühn^1^

^1^Division of Cancer Epidemiology, German Cancer Research Center (DKFZ), Heidelberg, Germany. ^2^University of Heidelberg Medical School, Heidelberg, Germany. ^3^Department of Neurology, University of Heidelberg, Heidelberg, Germany. ^4^Institute of Transfusion Medicine and Immunology, Heidelberg University, Medical Faculty Mannheim, and German Red Cross Blood Service Baden-Württemberg-Hessen, Mannheim, Germany

# Supplementary Figure 1. Flowchart Study Population

**
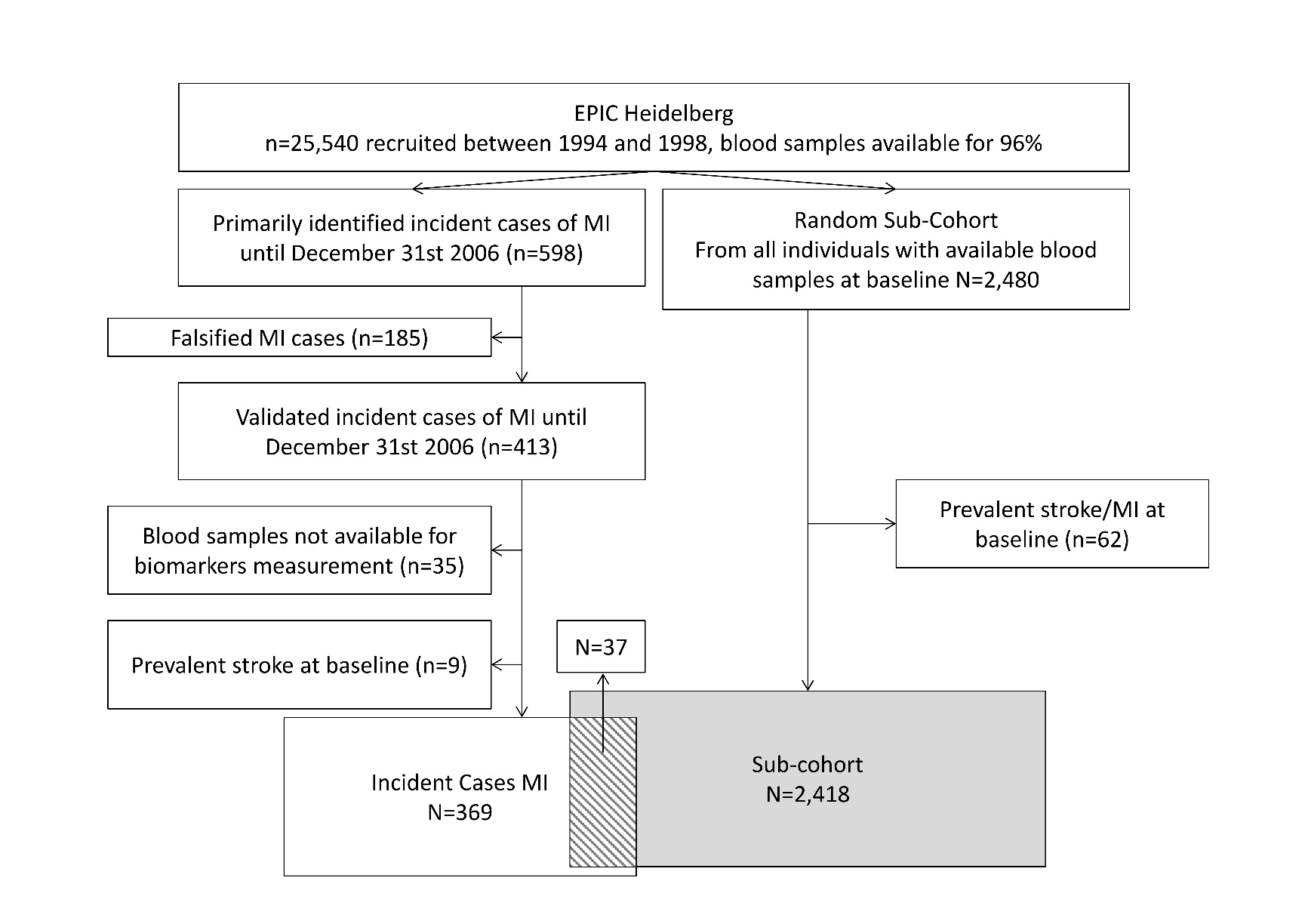
**
